# Elephant APOBEC3A cytidine deaminase induces massive double-stranded DNA breaks and apoptosis

**DOI:** 10.1038/s41598-018-37305-z

**Published:** 2019-01-24

**Authors:** Xiongxiong Li, Vincent Caval, Simon Wain-Hobson, Jean-Pierre Vartanian

**Affiliations:** 1 Molecular Retrovirology Unit, Institut Pasteur, CNRS-URA 3015, 28 rue du Docteur Roux, 75724 Paris, France; 2 Lanzhou Institute of Biological Products Co., Ltd (LIBP), subsidiary company of China National Biotec Group Company Limited (CNBG), 730046 Lanzhou, China

## Abstract

The incidence of developing cancer should increase with the body mass, yet is not the case, a conundrum referred to as Peto’s paradox. Elephants have a lower incidence of cancer suggesting that these animals have probably evolved different ways to protect themselves against the disease. The paradox is worth revisiting with the realization that most mammals encode an endogenous APOBEC3 cytidine deaminase capable of mutating single stranded DNA. Indeed, the mutagenic activity of some APOBEC3 enzymes has been shown to introduce somatic mutations into genomic DNA. These enzymes are now recognized as causal agent responsible for the accumulation of CG- > TA transitions and DNA breaks leading to chromosomal rearrangements in human cancer genomes. Here, we identified an elephant *A3Z1* gene, related to human *APOBEC3A* and showed that it could efficiently deaminate cytidine, 5-methylcytidine and produce DNA breaks leading to massive apoptosis, similar to other mammalian APOBEC3A enzymes where body mass varies by up to four orders of magnitude. Consequently, it could be considered that *eAZ1* might contribute to cancer in elephants in a manner similar to their proposed role in humans. If so, *eAZ1* might be particularly well regulated to counter Peto’s paradox.

## Introduction

The *APOBEC3 (A3)* locus is bounded by two conserved genes, *chromobox 6* and *7* (*CBX6* and *CBX7*) in most placental mammals and encodes a family of cytidine deaminases capable of converting cytidine residues to uridine in single strand DNA (ssDNA). The mutagenic activity of these enzymes is involved with the restriction of retroviruses and DNA viruses, as well as endogenous retroelements and retrotransposons through hypermutation of viral DNA in a process called editing^1^. The A3 repertoire is extremely variable among mammals, the locus being shaped through extensive gene duplications and functionalization in the context of a virus-host arms race. A3 enzymes are made up of three related, but distinct zinc-finger domains referred to as Z1, Z2 and Z3^2–4^ presumably already present in the genome of the ancestor of placental mammals^5^.

The last few years has seen the identification of two human endogenous A3 cytidine deaminases, APOBEC3A (A3A) and APOBEC3B (A3B) capable of introducing multiple mutations in chromosomal DNA^6–9^. These findings are grounded by the analysis of many cancer genomes, revealing far more mutations and rearrangements than hitherto imagined, where CG- > TA transitions appears to be the dominant mutations^10–13^.

Human A3A is composed of a single Z1 domain, while A3B is composed of a double Z2Z1 domain, although only the C terminal Z1 domain being catalytically functional^6^. A3A and A3B enzymes are both localized in the nucleus and can edit cytidine residues to uridine in ssDNA during transcription and replication, following DNA repair, and leave TpC to TpT signature mutations that show up in cancer genomes^6,8,9^. Both enzymes can mutate 5-methylcytidine (5MeC) to thymidine leaving another distinct signature in cancer genomes^6,14–16^.

Although A3A and A3B are accepted as intrinsic mutators of cellular chromosomal DNA, analyzed in several cancer types^8,11,17^, debate still persists regarding the contribution of each enzyme in the accumulation of mutations paving the way for oncogenesis. While, it has been described that A3A and A3B could be enzymatically active in different cancers^18^, A3A is the more active of the two enzymes and as a consequence, only A3A can produce double stranded breaks (DSBs), at least in an experimental setting^6,7,19^. Editing frequencies of >0.5 can be found which is why the phenomenon is referred to as hyperediting or hypermutation^9^. Accumulation of substitutions localized in the A3B C-terminal domain attenuated the activity of the enzyme compared to A3A^6^. Interestingly, this functional attenuation was also observed for the rhesus monkey rhA3B enzyme compared to rhA3A indicating that this mutagenic dichotomy was maintained for ~38 million years^6^. Moreover, the deletion of most of the *A3B* gene results in a higher odds ratio of developing breast, ovarian or liver cancer^20–23^. Indeed, complete genome sequencing of *ΔA3B*^*-/-*^ breast cancer genomes revealed a higher mutation burden^24^. Finally, fine analysis of signatures mutations in cancer genomes unraveled for twice as many A3A specific mutational signature (YTCA) over A3B (RTCA) suggesting a major role of A3A in cancer mutagenesis^8,25^.

Another difference between A3A and A3B lies in their evolutionary history. *A3A* is present across most placental mammals, indicating that this evolutionary experiment has been running ~150 million years^26^. There are some notable exceptions – an *A3A* gene is absent among all members of the order *Rodentia*, pigs, while for *Felidae* the gene is inactivated but identifiable^3,26^. By contrast *A3B* is unique to the order *Primates* and arose by gene conversion involving *A3A*. We have previously shown that the A3A enzymes from 8 mammalian species from rabbits to cows and horses were capable of deaminating C and 5MeC in ssDNA as well as producing DSBs, even though activities varied considerably^26^.

The incidence of developing cancer was hypothesized to increase with the body size, referred to as Peto’s paradox^27^. However, as large animals exist and do not invariably die of cancer this paradox fails to explain the presence of compensatory mechanisms that protect the genome. With this in mind, we were intrigued by a recent report showing that elephants appeared to have a lower-than-expected rate of cancer which might possibly be coupled to multiple copies of *TP53* even though most were processed pseudogenes^28,29^. It is equally possible that the A3 enzymes of large mammals could have been attenuated by mutation. Accordingly, we decided to explore the function of the elephant A3Z1 enzyme.

## Results and Discussion

### Synthesis and expression of elephant APOBEC3Z1 cytidine deaminase

**Figure 1:**
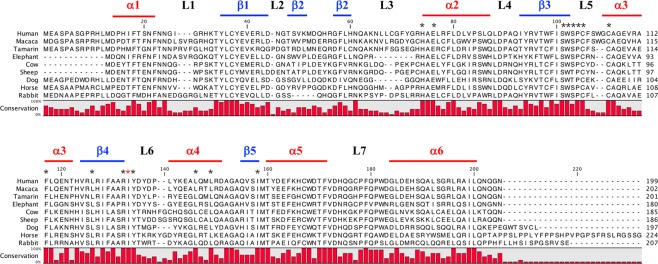
Comparison of APOBEC3Z1 cytidine deaminases. CLUSTALW alignment of A3A proteins. Sequence conservation is depicted in red for each residue. Asterisks represent residues involved in zinc coordination responsible for enzymatic activity. Red asterisk represents the isoleucine amino-acid specific to Z1 domain. Structural motifs structures (α helix, β sheet and loop) are indicated.

**Figure 2:**
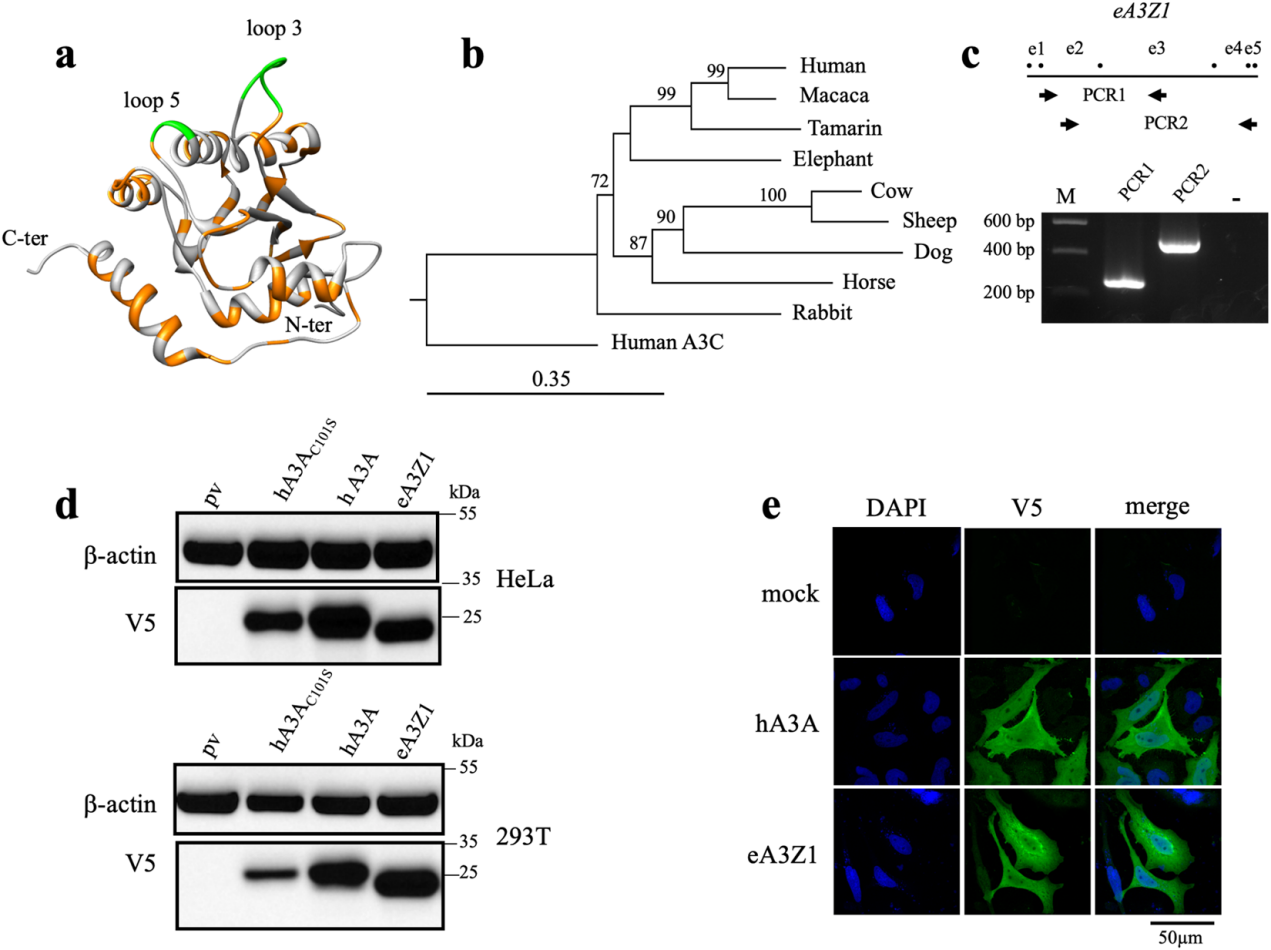
Elephant APOBEC3Z1 enzyme. (**a**) A3A structure with differences between human and elephant A3A presented in orange, loops 3 and 5 in green are absent in eA3Z1. (**b**) Phylogenetic tree of elephant A3A along with other mammalian enzymes. A3A protein sequences constructed using the Neighbor-joining method with the CLC Main Workbench 7.0.2 software. Human A3C was used as outlier. Numbers correspond to bootstrap values inferred from 1,000 replicates. Bootstrap values below the threshold of 70% are not shown. (**c**) Nested RT-PCR amplification of *eA3Z1* transcript in liver from an *African savana* elephant. PCR1 corresponds to the amplification of *eA3Z1* exon 2 to exon 3 and PCR2 corresponds to the amplification of exon 3 to exon 5; –:negative PCR control; M: molecular weight marker; e: exon. (**d**) Western blot detection of V5-tagged A3A proteins of human and elephant in HeLa and HEK-293T cells, pv (plasmid vector) and hA3A_C101S_ were used as negative controls. β-actin probing was used as loading control. (**e**) Confocal microscopy analysis of V5-tagged human and elephant A3A proteins in HeLa at 48 hours post transfection. Nuclei are stained with DAPI.

To explore the implication of elephant A3 enzyme in tumorigenesis, *in silico* data mining was performed using blast/blat analyses of the genomic for *A3Z1* like sequences. We retrieved an elephant *A3Z1* sequence named *eA3Z1*, equivalent to the p2 protein form of human A3A (hA3A), that is to say missing the first coding exon, what is also a feature of dog, horse and cow *A3A* genes. Furthermore, p1 and p2 forms of A3A are functionally equivalent^26,30,31^. There was 44% amino acid divergence between the human and elephant protein sequences (Fig. 1). The elephant sequence carried an 8 residue deletion in loop 3, which is not without precedent^26^ and impacts little the overall structure as can be seen from Fig. 2a. All the key amino acid residues typical of an A3A enzyme were conserved^3^. A phylogenetic analysis using the neighbor-joining method revealed that eA3Z1 was closely related to those from the *Primate* lineage (Fig. 2b). To prove that eA3Z1 is expressed *in vivo* and to validate the putative *eA3Z1* DNA sequence inferred from elephant genome assembly, total RNA from the liver of an African Savana elephant (*Loxodonta africana*) that had died from an encephalomyocarditis virus infection^32^ was analyzed. Total RNA was extracted, cDNA generated and sequences corresponding to complete *eA3Z1* transcripts were amplified by a semi-nested PCR procedure. As shown in Fig. 2c, strong *eA3Z1* cDNA amplifications were obtained giving rise to two overlapping PCR products. Finally, *eA3Z1* sequence (accession number: MK156802) was validated by direct sequencing (Supplementary Fig. S1) and identical to the previous BLAST/BLAT analysis search sequences from genomic data.

Accordingly, an e*A3Z1* cDNA was synthesized and cloned into pcDNA3.1D/V5-His-TOPO with a strong Kozak motif (ACCATG) for functional studies. When overexpressed in transfected HeLa or HEK-293T cells, Western blot analysis revealed a strong expression of V5-tagged eA3Z1 on a par with that for its human counterpart (hA3A) or an inactive mutant (hA3A_C101S_; Fig. 2d). The slightly lower molecular weight of eA3Z1 is in agreement with the calculated molecular weights (hA3A 23.0 kDa; eA3Z1 20.9 kDa). The subcellular localization was assessed in HeLa cells by confocal microscopy. Anti-V5 staining revealed that eA3Z1 exhibited the classical nuclear and cytoplasmic distribution described for human (Fig. 2e) as well as other mammalian A3A enzymes^26^.

### Elephant APOBEC3Z1 editing of nuclear DNA and 5-methylcytidine

**Figure 3:**
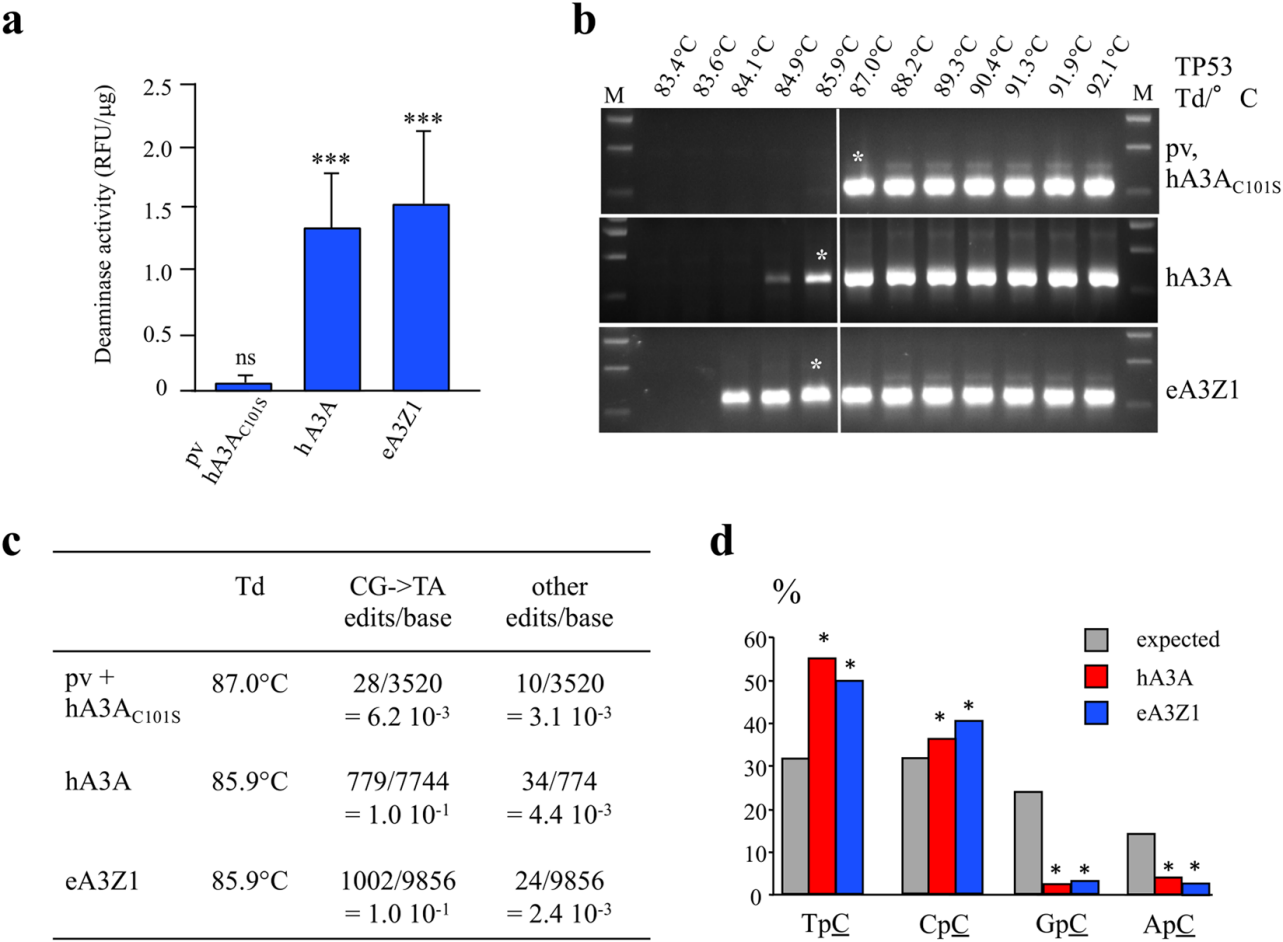
Editing of *TP53* with human and elephant APOBEC3A. (**a**) Fluorescence resonance energy transfer assay (FRET)-based *in vitro* deamination assay of hA3A and eA3Z1 performed on FAM-TAMRA coupled oligonucleotides using transfected HEK-293T lysates. Pv and hA3A_C101S_ were used as negative controls. Results are expressed in Relative Fluorescence Unit per μg of protein (RFU/μg). Differences were calculated using the Unpaired t test (***p < 0.001, ns: no significance). (**b**) 3D-PCR recovered edited TP53 DNA down to 84.9°C and 84.1°C for hA3A and eA3Z1 respectively. The white line indicates the threshold between edited and unedited 3D-PCR products in terms of the denaturation temperature. Pv and hA3A_C101S_ showed no editing of TP53 and were used as negative control. Asterisks refer to the samples cloned and sequenced. M, molecular weight markers. (**c**) CG- > TA mutation frequencies analyzed with hA3A_C101S_ and pv at 87°C and with hA3A and eA3Z1 at 85.9°C. (**d**) Dinucleotide context of *TP53* DNA region on minus strand DNA obtained with hA3A and eA3Z1. Chi-square test indicates dinucleotide frequencies that significantly deviate from the expected values (*p < 0.05).

To assay catalytic activity, *eA3Z1* and *hA3A* plasmids were transfected into HEK-293T cells and cellular lysates were used in a fluorescence resonance energy transfer assay based *in vitro* deamination assay where C to U conversion in a TAMRA-FAM-labeled DNA oligonucleotide allows fluorescence detection following cleavage by uracil-DNA glycosylase (UNG) activity^7^. Elephant eA3Z1 activity was on a par with hA3A (Fig. 3a). To explore A3 hyperediting of chromosomal DNA, the HEK-293T-UGI cell line was transfected with *eA3Z1* and *hA3A* plasmids. The HEK-293T-UGI cell line constitutively expresses uracil N-glycosylase (UNG) inhibitor (UGI) where UNG is the crucial enzyme involved in excising uracil from DNA. As UNG is rate limiting for the detection of hyperedited chromosomal DNA, inhibition by UGI is necessary^9^. At 48 hours post-transfection, total DNA was extracted and *TP53* DNA was amplified by 3D-PCR, a technique that selectively amplifies A3-edited ssDNA molecules^33^. The lowest PCR denaturation temperature (Td) allowing amplification of unedited *TP53* target DNA was 87°C (Fig. 3b). For *eA3Z1* and *hA3A* transfections, 3D-PCR products were recovered at Tds as low as 84.1°C and 84.9°C respectively which is diagnostic for A3 editing (Fig. 3b). Nonetheless, 3D-PCR products from the 85.9°C amplification (white asterisk, Fig. 3b) were cloned and sequenced as those of the last positive amplification for plasmid vector (pv) and hA3A_C101S_ as negative controls.

Hyperedited *TP53* target sequences were recovered with an average editing frequency of 10% compared to a background value of 0.6% (Fig. 3c). The monotony of editing is confirmed by the frequency of non-CG- > TA mutations which did not differ from background values. Cytidine editing was strongly associated with TpC, and to a lesser extent CpC dinucleotides (Fig. 3d) to the detriment of GpC and ApC which is typical for mammalian A3A enzymes^26,34–37^.

To demonstrate that no endogenous activity of hA3A present in HEK-293T-UGI cells would give rise to hypermutated sequences, hA3A or eA3Z1 transfections were performed in QT6 cell lines in presence of UGI. QT6 is a quail cell line and was chosen as there is no endogenous A3 background^38^. As expected, 3D-PCR product sequence analysis demonstrated the same profile of hypermutated sequences and dinucleotide contexts (data not shown).

**Figure 4:**
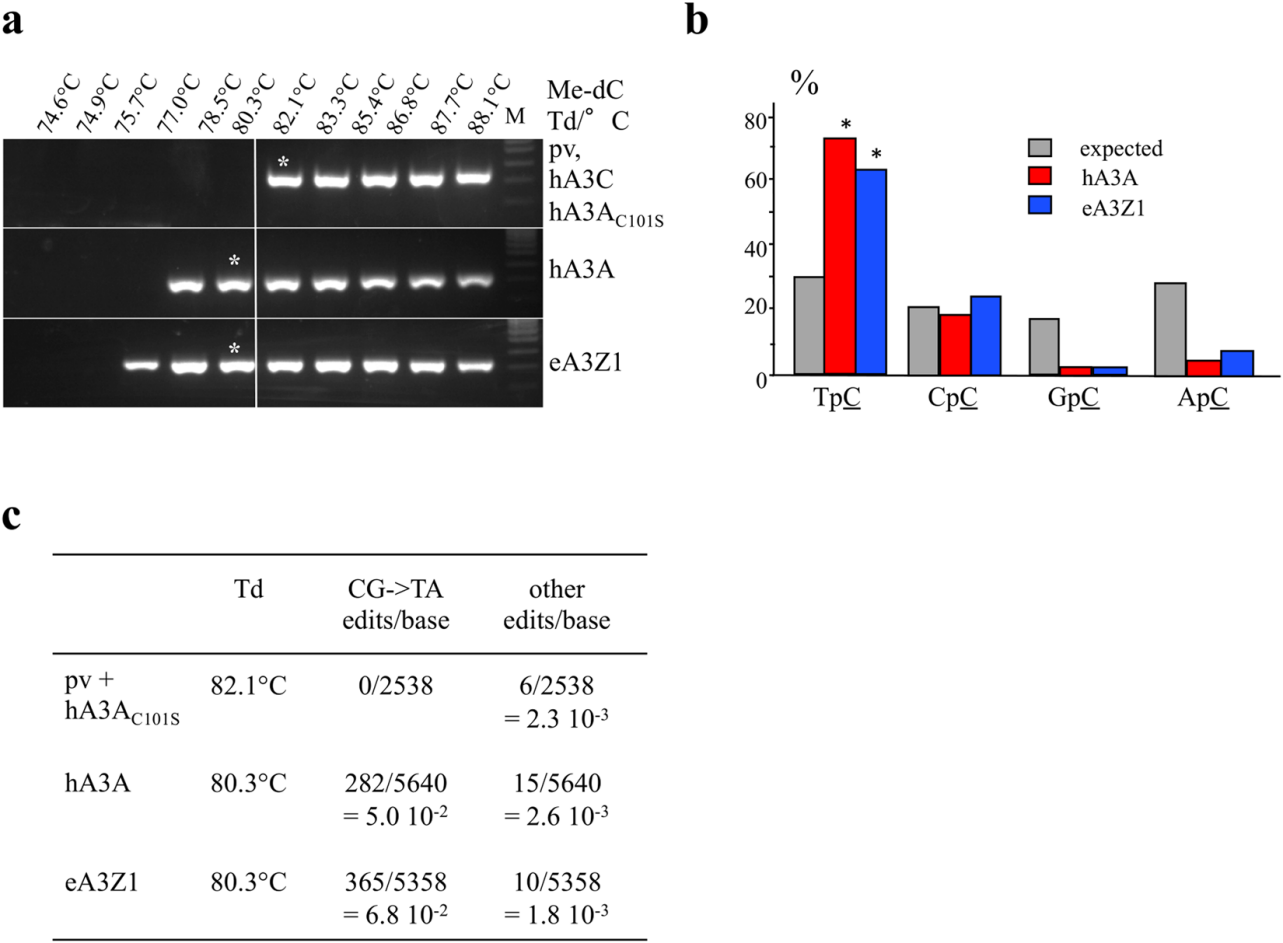
Elephant APOBEC3Z1 deamination of 5-methylcytidine. (**a**) 3D-PCR recovered edited 5MeC substituted HIV env DNA down to 77°C and 75.7°C for hA3A and eA3Z1 respectively. The white line indicates the threshold between edited and unedited 3D-PCR products in terms of the denaturation temperature. Pv and hA3A_C101S_ showed no editing of 5MeC substituted HIV *env* DNA and were used as negative control. Asterisks refer to the samples cloned and sequenced. M, molecular weight markers. (**b**) Dinucleotide context for human and elephant A3A deamination of 5MeC. Chi-square test indicates dinucleotide frequencies that significantly deviate from the expected values (*p < 0.05). (**c**) CG- > TA mutation frequencies analyzed with hA3A_C101S_ and pv at 82.1°C and with hA3A and eA3Z1 at 80.3°C.

One of the singular traits of mammalian A3A deaminases is their ability to efficiently deaminate 5MeC^26^. To demonstrate that eA3Z1 can deaminate 5MeC, QT6 cells were transfected with the eA3Z1 expression plasmid and subsequently transfected by 5MeC-substituted HIV *env* DNA fragments^16^. As shown in Fig. 4a, 3D-PCR products were recovered at temperatures as low as 75.7°C and 77 °C for the eA3Z1 and hA3A transfections respectively, compared to 82.1°C for the plasmid vector or hA3A_C101S_ (Fig. 4a). The 82.1°C and 80.3°C 3D-PCR products (Fig. 4a), indicated by an asterisk were cloned, sequenced and confirmed the presence of edited 5MeC in the expected TpC dinucleotide context (Fig. 4b,c). Hyperedited 5MeC V1V2 target sequences were recovered with an average editing frequency of 5% and 6.8% respectively for hA3A and eA3Z1 compared to background value (Fig. 4c).

### Elephant APOBEC3Z1 induces double strand DNA breaks and apoptosis

**Figure 5:**
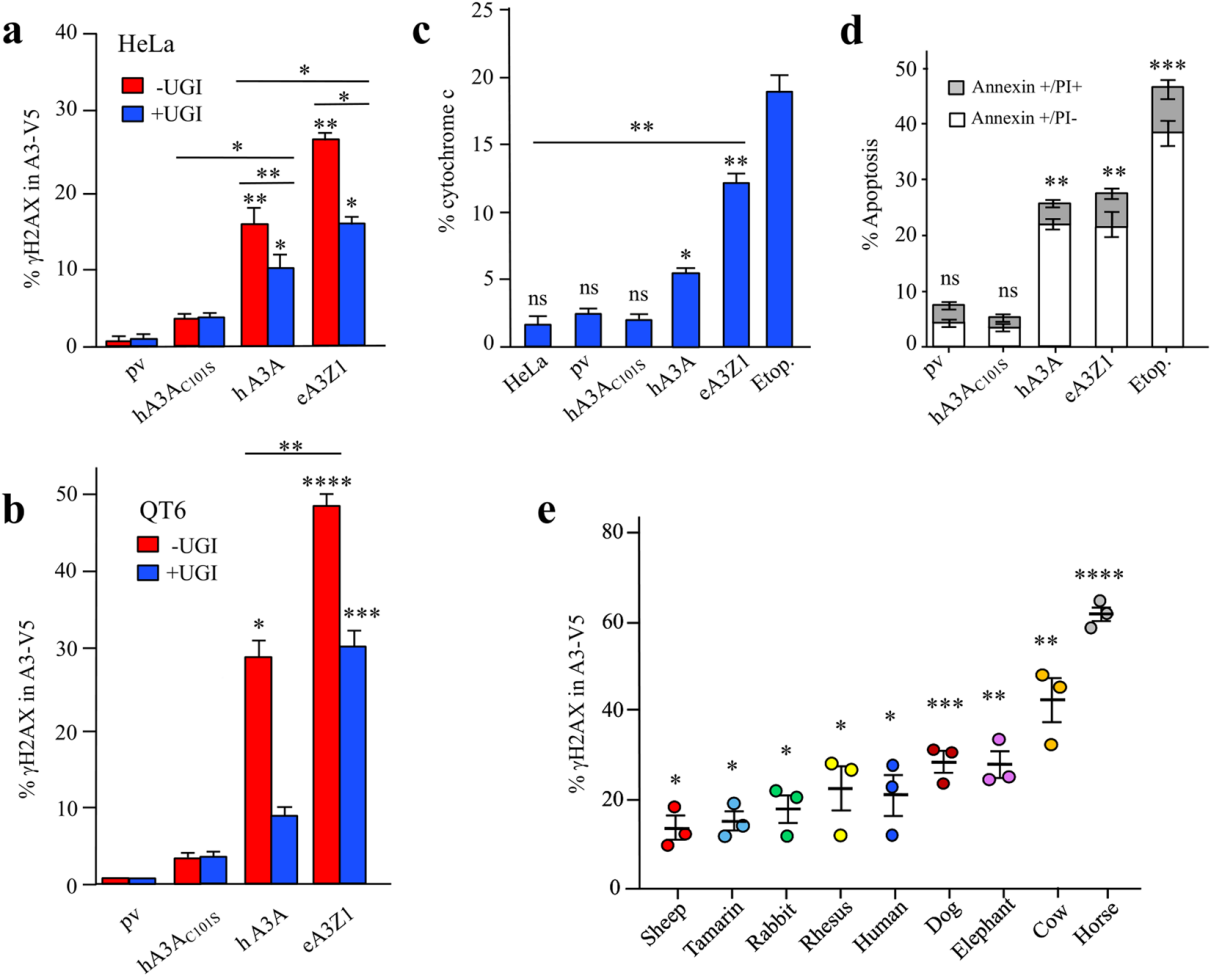
APOBEC3Z1 proteins can induce DSBs and apoptosis. (**a**,**b**) FACS analysis of γH2AX-positive HeLa and QT6 cells at 48 hours post transfection with pv, hA3A, eA3Z1 in presence or absence of UGI. Percentage of γH2AX positive cells are gated on V5 positive cells except for mock and UGI transfections. Error bars represent s.d. from three independent transfections. Differences were calculated using the Unpaired t test (*p < 0.05, **p < 0.01, ***p < 0.001, ****p < 0.0001). (**c**) FACS analysis of cytochrome c release in transfected cells with pv, hA3A_C101S_, hA3A, eA3Z1 and etoposide used as positive control. Differences were calculated using the Unpaired t test (*p < 0.05, **p < 0.01, ns: no significance). (**d**) FACS analysis of early apoptosis and late apoptosis/necrosis in HeLa cells transfected with pv, hA3A_C101S_, hA3A, eA3Z1 and etoposide used as positive control. Early apoptosis (Annexin V-positive, PI-negative cells, white) and late apoptosis/necrosis (Annexin V-positive, PI double-positive cells, grey) were analyzed at 48 hours post-transfection. Error bars represent s.d. from three independent transfections. Top error bars correlate with Annexin staining, whereas lower error bars represent s.d. for PI staining among Annexin + cells. Differences were calculated using the Unpaired t test (**p < 0.01, ***p < 0.001, ns: no significance). (**e**) FACS analysis of γH2AX-positive HeLa cells gated on the V5-tagged A3A from different animal species. Differences were calculated using the Unpaired t test (*p < 0.05, **p < 0.01, ***p < 0.001, ****p < 0.0001).

Human A3A editing of chromosomal DNA results in the formation of DSBs and can be readily scored by analysis of histone variant H2AX phosphorylation at serine 139 (γH2AX), a well-known marker for DSBs and the DNA damage response^39^. HeLa and QT6 cell lines were transfected with eA3Z1 and hA3A constructs ±UGI using plasmid vector as negative control. As can be seen in Fig. 5a, eA3Z1 and hA3A generated DSBs in HeLa cells ∼35 and ∼20-fold over background. While DSBs were more pronounced in QT6 with ∼70 and ∼40-fold higher over plasmid control with eA3Z1 being the more active of the two enzymes (Fig. 5b). In the presence of UGI, a decrease in A3-induced DSBs was noted for hA3A and eA3Z1 transfected cells indicating that UNG plays an important role in the formation of DSBs upon DNA editing. As A3-induced DSBs lead to apoptosis, we measured cytochrome c release (Fig. 5c). Transfection of equal amounts of plasmid DNA resulted in greater cytochrome c release for eA3Z1 using Annexin V and propidium iodide as markers for apoptosis (Fig. 5c,d). These data demonstrate that eA3Z1 is a strong editor of ssDNA and can induce DSB leading to apoptosis.

Taken together, these data demonstrate that eA3Z1 clearly exhibit an enzymatic activity similar to human and other mammalian A3A cytidine deaminases since eA3Z1 edits both C and 5MeC residues in ssDNA and can make DSBs leading to apoptosis. A side-by-side comparison of eA3Z1 to 8 mammalian A3As is shown in Fig. 5e. All constructs are well expressed by Western blotting and immunofluorescence with the signal exception of the tamarin construct^26^. While eA3Z1 is well ranked among the series, it is less efficient than the cow and horse constructs which, although they have large body mass and longevity (wild cattle 18–25 + years, up to 900 kg; horses 30–40 years, up to 600 kg), are not comparable to the elephant (median 56 years, up to 70 years, up to 7000 kg). Obviously, there are many variables that can alter A3 function: mi- and lncRNAs and transcription factor sites in the promoter as well as the role of negative interactors like TRIB3^34,40^ which is part of the broad CtIP-Rb-BRCA1-ATM protein network that involves cell cycle control, cell survival, DNA repair, and genome stability.

The eA3Z1 described here is clearly comparable to those of many large mammals, being able to damage chromosomal DNA and might therefore contribute to oncogenesis. If so, perhaps the A3s of elephants must be tightly regulated to lower the incidence of cancer.

## Methods

### Plasmids and samples

Elephant *A3Z1* cDNAs were synthetized (GeneCust) and subsequently cloned into pcDNA3.1/V5-His-TOPO vector (Invitrogen). All constructs were C-tagged by the V5 epitope. Catalytically inactive hA3A mutants, UGI expression plasmid and other mammalian A3A expression plasmids, sheep, tamarin, rabbit, rhesus monkeys, dog, cow and horse were already described. All plasmids were verified by sequencing. A fatal case of encephalomyocarditis virus involving an African elephant (*Loxodonta africana*) occurred in November 2013 at the Réserve Africaine de Sigean in France^32^. Naturally infected samples were collected as part of routine veterinary investigation carried out by qualified veterinarians in the area of origin. All methods were carried out in accordance with relevant guidelines and regulations.

### Cell transfection

Approximately 800,000 HeLa, HEK-293T, HEK-293T-UGI and QT6 cells were seeded into 6-well plates and transfected with 2 µg of A3 plasmid using the jetPRIME transfection kit (Polypus Transfection™) according to manufacturer’s instructions. For cotransfections, a plasmid ratio of 1:1 was used.

### Deamination assay

At 48 hours post-transfection, A3-transfected 293 T cells were extensively washed with PBS and mechanically harvested. Total proteins were extracted using specific lysis buffer (25 mM HEPES pH 7.4, 10% glycerol, 150 mM NaCl, 0.5% Triton X-100, 1 mM EDTA, 1 mM MgCl_2_, 1 mM ZnCl_2_) supplemented with protease inhibitors. Deaminase activity was assessed by incubating whole cell lysates with 1 pmole DNA oligonucleotide 5′FAM-AAATTCTAATAGAT AATGTGA-TAMRA in the presence of 0.4 unit of uracil-DNA-glycosylase (UDG) (New England Biolabs) in a 20 mM Tris-HCl, 1 mM dithiothreitol, and 1 mM EDTA reaction buffer. After 2 hours of incubation at 37 °C, abasic sites were cleaved by heating for 2 min at 95 °C and end point fluorescence was measured using a RealPlex^2^ Mastercycler (Bio-Rad) with FAM setting and background fluorescence obtained with mock-transfected cells as negative control. Results are normalized to the quantity of protein using Pierce BCA protein assay kit (Thermo Scientific).

### RNA and DNA extraction, 3D-PCR amplification and cloning

Total RNA from the elephant liver and DNA from transfected cells were extracted using the MasterPure^TM^ complete DNA and RNA purification kit (Epicentre) and suspended in 35 µL of sterile water. cDNAs were synthetized using QuantiTect reverse transcription kit (Qiagen). For *eA3Z1* amplification, a semi nested PCR was performed. For PCR1 and PCR2, first round primers were 1MYfwd: 5′CTGATGGATCAAAACATATTCCGCTTCA and 2MYrev: 5′TCAGTTGTTTCCATTCTGGAGAATAC. Second round primers for PCR1 were, 1MYfwd: 5′CTGATGGATCAAAACATATTCCGCTTCA and 1MYrev: 5′TGGGCACAGTTACGGCAGGGACTC. And for PCR2, second round primers were: 2MYfwd: 5′GGCCAGAAACAGACCTACCTGTGC and 2MYrev: 5′TCAGTTGTTTCCATTCTGGAGAATAC. First and second round reaction parameters were 95 °C for 5 min, followed by 35 cycles of 95 °C for 30 sec., 60 °C for 30 sec. and 72 °C for 1 min., with final extension for 10 min. at 72 °C. PCR1 and 2 products direct sequencing were outsourced to Eurofins and performed using amplification primers: 1MYfwd, 1MYrev, 2MYfwd and 2MYrev (accession number: MK156802).

For *TP53* amplification, primers were: TP53out 5′GAGCTGGACCTTAGGCTCCAGAAAGGACAA TP53outrev 5′GCTGGTGTTG TTGGGCAGTGCTAGGAA, amplification was performed using first-round standard PCR with 5 µL of DNA extract followed by nested 3D-PCR^9,33^ with 5 µL of 1/50 dilution of the first PCR round. Primers for nested 3D-PCR were: TP53in 5′TTCTCTTTTCCTATCCTGAGTAGTGGTAA and TP53inrev 5′AAAGGTGATAAAAGTGAATCTGAGGCATAA. 3D-PCR was performed in 50 µL with 1 U Taq DNA polymerase (Eurobio) per reaction. PCR conditions for the first round of amplification were 5 min. of denaturation at 95 °C then 40 cycles of amplification (30 sec. 95 °C, 30 sec. 58 °C, 30 sec. 72 °C), followed by 7 min. at 72 °C. The condition of 3D-PCR were 5 min of denaturation temperature gradient of 84–92 °C then 40 cycles of amplification (1 min. 84–92 °C, 30 sec. 58 °C, 2 min. 72 °C), followed by 10 min. at 72 °C.

For the 5MeC deamination assay, a 679 bp fragment of HIV-I LAI *env* gene was amplified using total substitution of dCTP by 5Me-dCTP (Trilink) using the primer pair MC1, 5′TTGATGATCTGTAGTGCTACAGCA and MC2, 5′GCCTAATTCCATGTGTACATTGTA. The 5MeC containing DNA was heat denatured and chilled on ice and 200 ng of synthesized DNA was transfected using jetPRIME 24 hours following initial transfection of A3 coding plasmids in QT6 cells as described earlier^16^. The second round PCR was classical PCR, primers were: MC3 5′TGTACCCACAGACCCCAACCCACAA and MC4 5′TTCCATTGAACGTCTTATTATTACA, and PCR parameters were: 95 °C for 5 min., followed by 30 cycles of amplification (45 sec. 95 °C, 45 sec. 54 °C, 90 sec. 72 °C) followed by 20 min. at 72 °C. The 3D-PCR reaction parameters were 75–88 °C for 5 min., followed by 35 cycles of amplification (45 sec. 75–88 °C, 45 sec. 56 °C, 90 sec. 72 °C) followed by 20 min. at 72 °C, primers were MC5, 5′ATCAAAGCCTAAAGCCATGTGTAA and MC6, 5′CAATAATGTATGGGAATTGGCTCAA. 3DPCR products were cloned into the pCR2.1 TOPO cloning vector (Invitrogen) and sequenced (Eurofins).

### Immunofluorescence

Approximately 50,000 HeLa cells were seeded in Nunc™ Lab-Tek™ II Chamber Slide™ System Thermo Scientific™ and transfected 24 hours later with 1 µg of plasmid DNA according to the Fugene® protocol. Two days after transfection, coverslip grown transfected HeLa cells were washed three times with PBS and fixed with 4% paraformaldehyde (Electron Microscopy Sciences) for 15 min. Cells were then washed two times and permeabilized with a 50% methanol/acetone mix for 10 min. After two PBS washings, permeabilized cells were incubated for 1 hour at room temperature, first with 0.5% bovine serum albumin (BSA) PBS 1/200 mouse monoclonal anti-V5 antibody (Invitrogen) and then with 0.5% bovine serum albumin PBS 1/500 anti mouse Alexa Fluor 488 conjugated antibody (ThermoFisher). After several PBS washings, coverslips were mounted with Vectashield mounting medium for immunofluorescence (Interchim). Imaging was performed using a Leica SP5 confocal microscope.

### FACS analysis, double strand-breaks and apoptosis

Transfected cells were trypsinized, washed with PBS, fixed in 2% ice-cold paraformaldehyde (Electron Microscopy Sciences) for 10–20 min. on ice. After one PBS washing, cells were permeabilized in 90% ice-cold methanol (Sigma) for 30 min. For DSBs experiments, fixed and permeabilized cells were incubated 1 hour on ice with 1:200 PBS-0.5% BSA diluted mouse monoclonal anti V5-Tag Alexa Fluor® 488 antibody (AbD Serotec) and 1:100 diluted Alexa Fluor® 647 Mouse anti-H2AX (BD Pharmingen). For apoptosis, transfected HeLa cells were collected and washed with PBS, then incubated with complete DMEM medium at 37 °C for 2 hours. After washing with cold PBS, cells were resuspended in 1X Binding Buffer (BD Pharmingen) and then counterstained with 1 μg/ml FITC Annexin V antibody (BD Pharmingen) and 5 μg/ml Propidium Iodide (PI) (BD Pharmingen) to distinguish between early apoptotic and late apoptotic or necrotic events. Treatment by 100 mM etoposide in dimethylsulfoxide was used as positive control. The labelled samples were analyzed on a MACSQuant® analyzer harboring violet, blue, and either a red laser (measure of dsDNA breaks and apoptosis). The data were analyzed using the FlowJo® software (Tree Star Inc., version 10.1r5 for Mac).

### Mitochondrial cytochrome c release

At 48 hours post-transfection, HeLa cells were trypsinized and investigated for cytochrome c release by using the FlowCellect cytochrome c kit from Millipore according to manufacturer’s instructions. Cells treated with 200 µM etoposide for 16 hours were used as a positive control of cytochrome c release. Stained samples were acquired on a MACSQuant Analyzer (Miltenyi Biotec) and the data was analyzed with FlowJo software (Tree Star Inc. version 10.0.8). For each sample 10,000 cells were counted.

### Western blotting

Cells were recovered 48 hours after transfection. Protein extraction and Western blot analysis were carried out according to standard procedures. After blocking, membranes were probed with either a 1:5000 dilution of anti V5-tag horseradish peroxidase-coupled antibody (Invitrogen), or a 1:15000 dilution of anti β-actin (Sigma). The membrane was subjected to detection by SuperSignal™ West Pico chemiluminescent substrate (ThermoFisher Scientific).

## Supplementary information

Supp. Figure S1
