# Supplementary material for: Elephant APOBEC3A cytidine deaminase induces massive double-stranded DNA breaks and apoptosis
Source: Sci Rep. 2019 Jan 24;9:728. doi: 10.1038/s41598-018-37305-z (PMC6345769; doi:10.1038/s41598-018-37305-z)
Supplement: Supplementary file 1 — Supp. Figure S1 [file 41598_2018_37305_MOESM1_ESM.docx]

-------------------------------------------------------------------------------------------------100

**eA3Z1 ctgATGGATCAAAACATATTCCGCTTCAACTTTATTAATGACGCTTCCGTCCGTGGCCAGAAACAGACCTACCTGTGCTATGAGGTGGAGCTCCTAGATGGCA**

**<M><D><Q><N><I><F><R><F><N><F><I><N><D><A><S><V><R><G><Q><K><Q><T><Y><L><C><Y><E><V><E><L><L><D><G><**

eA3Z1_1fwd.ab1 ATGGATCAAAACATATTCCGCTTCAACTTTATTAATGACGCTTCCGTCCGTGGCCAGAAACAGACCTACCTGTGCTATGAGGTGGAGCTCCTAGATGGCA

eA3Z1_1rev.ab1 ---------------------------------------------------GGCCAGAAACAGACCTACCTGTGCTATGAGGTGGAGCTCCTAGATGGCA

eA3Z1_2fwd.ab1 -------------------------------------------------------------------ACCTGTGCTATGAGGTGGAGCTCCTAGATGGCA

eA3Z1_2rev.ab1 -------------------------------------------------------------------------------------------TAGATGGCA

-------------------------------------------------------------------------------------------------200

**eA3Z1 ACTCCTGGGTCCCGTTGGATGAGGGCAGGGGCTTCCTCCTTAATCAGCCACGCCGCCATGCAGAGTTGTGCTTCCTGGACCGGGTTTCTTCTTGGCATCT**

**N><S><W><V><P><L><D><E><G><R><G><F><L><L><N><Q><P><R><R><H><A><E><L><C><F><L><D><R><V><S><S><W><H><L**

eA3Z1_1fwd.ab1 ACTCCTGGGTCCCGTTGGATGAGGGCAGGGGCTTCCTCCTTAATCAGCCACGCCGCCATGCAGAGTTGTGCTTCCTGGACCGGGTTTCTTCTTGGCATCT

eA3Z1_1rev.ab1 ACTCCTGGGTCCCGTTGGATGAGGGCAGGGGCTTCCTCCTTAATCAGCCACGCCGCCATGCAGAGTTGTGCTTCCTGGACCGGGTTTCTTCTTGGCATCT

eA3Z1_2fwd.ab1 ACTCCTGGGTCCCGTTGGATGAGGGCAGGGGCTTCCTCCTTAATCAGCCACGCCGCCATGCAGAGTTGTGCTTCCTGGACCGGGTTTCTTCTTGGCATCT

eA3Z1_2rev.ab1 ACTCCTGGGTCCCGTTGGATGAGGGCAGGGGCTTCCTCCTTAATCAGCCACGCCGCCATGCAGAGTTGTGCTTCCTGGACCGGGTTTCTTCTTGGCATCT

-------------------------------------------------------------------------------------------------300

**eA3Z1 GGACCCCACGAAGCACTACAAATTCACCTGGTTCCTTTCCTGGAGTCCCTGCCGTAACTGTGCCCAGGAAGTGGTTGCCTTCCTGGGGGGGAATAGCCAC**

**><D><P><T><K><H><Y><K><F><T><W><F><L><S><W><S><P><C><R><N><C><A><Q><E><V><V><A><F><L><G><G><N><S><H>**

eA3Z1_1fwd.ab1 GGACCCCACGAAGCACTAC---------------------------------------------------------------------------------

eA3Z1_1rev.ab1 GGACCCCACGAAGCACTACAAATTCACCTGGTTCCTTTCCTGGAGTCCCTGCCGTAACTGTGCCCA----------------------------------

eA3Z1_2fwd.ab1 GGACCCCACGAAGCACTACAAATTCACCTGGTTCCTTTCCTGGAGTCCCTGCCGTAACTGTGCCCAGGAAGTGGTTGCCTTCCTGGGGGGGAATAGCCAC

eA3Z1_2rev.ab1 GGACCCCACGAAGCACTACAAATTCACCTGGTTCCTTTCCTGGAGTCCCTGCCGTAACTGTGCCCAGGAAGTGGTTGCCTTCCTGGGGGGGAATAGCCAC

-------------------------------------------------------------------------------------------------400

**eA3Z1 GTGAGCCTGAGCATCTTTGCCCCCCGAATCTATGATTACTACTCGGGATATGAGGAGGGCCTGCGCTCACTGCAGGGGGCCGGGGCCCATGTGTCCATCA**

**<V><S><L><S><I><F><A><P><R><I><Y><D><Y><Y><S><G><Y><E><E><G><L><R><S><L><Q><G><A><G><A><H><V><S><I><**

eA3Z1_1fwd.ab1 ----------------------------------------------------------------------------------------------------

eA3Z1_1rev.ab1 ----------------------------------------------------------------------------------------------------

eA3Z1_2fwd.ab1 GTGAGCCTGAGCATCTTTGCCCCCCGAATCTATGATTACTACTCGGGATATGAGGAGGGCCTGCGCTCACTGCAGGGGGCCGGGGCCCATGTGTCCATCA

eA3Z1_2rev.ab1 GTGAGCCTGAGCATCTTTGCCCCCCGAATCTATGATTACTACTCGGGATATGAGGAGGGCCTGCGCTCACTGCAGGGGGCCGGGGCCCATGTGTCCATCA

-------------------------------------------------------------------------------------------------500

**eA3Z1 TGACCTCTACAGAGTTTGAGCATTGCTGGAGAACCTTTGTAGACAACCGTGGATGTCCCTTTGTGCCCTGGAATAGGCTGGGCGAAAACAGCCAAACCAT**

**M><T><S><T><E><F><E><H><C><W><R><T><F><V><D><N><R><G><C><P><F><V><P><W><N><R><L><G><E><N><S><Q><T><I**

eA3Z1_1fwd.ab1 ----------------------------------------------------------------------------------------------------

eA3Z1_1rev.ab1 ----------------------------------------------------------------------------------------------------

eA3Z1_2fwd.ab1 TGACCTCTACAGAGTTTGAGCATTGCTGGAGAACCTTTGTAGACAACCGTGGATGTCCCTTTGTGCCCTGGAATAGGCTGGGCGAAAACAGCCAAACCAT

eA3Z1_2rev.ab1 TGACCTCTACAGAGTTTGAGCATTGCTGGAGAACCTTTGTAGACAACCGTGGATGTCCCTTTGTGCCCTGGAATAGGCTGGGCGAAAACAGCCAAACCAT

----------------------------------------540

**eA3Z1 ATCGAGGAGGCTGCAGAGTATTCTCCAGAATGGAAACAACtga**

**><S><R><R><L><Q><S><I><L><Q><N><G><N><N>**

eA3Z1_1fwd.ab1 ----------------------------------------

eA3Z1_1rev.ab1 ----------------------------------------

eA3Z1_2fwd.ab1 ATCG------------------------------------

eA3Z1_2rev.ab1 ATCGAGGAGGCTGCAGAGTATTCTCCAGAATGGAAACAACtga

Alignment of sequencing products from nested-PCR performed on elephant liver against *in silico* obtained eA3Z1 sequence. Sequences in blue correspond to PCR primers used for PCR1 and in red for PCR2.

**Elephant APOBEC3A cytidine deaminase induces massive double-stranded DNA breaks and apoptosis**

Xiong Xiong Li, Vincent Caval, Simon Wain-Hobson and Jean-Pierre Vartanian
